# Newborn thermal care in western Uttar Pradesh — gap analysis between knowledge and practices

**DOI:** 10.1186/s13690-022-00809-2

**Published:** 2022-02-16

**Authors:** Anurag Srivastava, Sumit Saxena, Payal Srivastava, Syed Esam Mahmood, Ruchi Pandey, Anju Saxena

**Affiliations:** 1 Government Institute of Medical Sciences, Greater Noida, India; 2 Autonomous State Medical College & Allied Pt Ram Prasad Bismil Memorial Hospital, Shahjahanpur, India; 3 College of Medicine, King Khalid University, Abha, Kingdom of Saudi Arabia; 4 Rohilkhand Medical College & Hospital, Bareilly, India

**Keywords:** Neonatal care, Thermal care, Neonatal care practices, Thermal care knowledge

## Abstract

**Background:**

The provision of health care services including maternal and newborn care is a dynamic system of entitlement and obligations among the community, the service providers, and the government. Thermal control remains poor in newborns owing to immaturity of the thermoregulatory center and newborn become vulnerable to hypothermia especially premature babies, intrauterine growth retardation and LBW babies, and even normal babies.This study aimed to assess the knowledge & practices regarding thermal protection their determinants.

**Methods:**

Cross-sectional study was conducted in the Amroha district. The study population comprised women of reproductive age (15 to 49 years) who have delivered a live baby within the past 12 weeks before the conduct of the study. Out of 6 blocks, 2 most populous villages were selected. Total 61 villages from 6 blocks were covered under the study. Knowledge and practices regarding newborn thermal care were expressed in percentages and compared.

**Results:**

The knowledge domain on thermal protection of baby, 60.9% of the respondents were well aware of how to keep baby warm after delivery, 71.4% of respondents knew that baby should be dried soon after birth, 64.9% of the respondents had an idea of time to dry the baby, 69.6% of the respondents knew that baby should be wrapped soon after birth.

**Conclusion:**

The findings of the study provides an insight into the existing knowledge and necessitate a need for quantitative studies in the study area to access knowledge & practices related to thermal protection of newborns. The authors emphasize a need for improving community awareness for the promotion of newborn care and improve the health system to meet the demands of birthing mothers and the needs of newborns.

**Supplementary Information:**

The online version contains supplementary material available at 10.1186/s13690-022-00809-2.

## Background

The provision of health care services including maternal and newborn care is considered to be a dynamic system of entitlement and obligations among people, the communities, service providers, and the concerned governments. The global commitment of Sustainable Development Goals (SDGs) as per target 3.2 to “end all the preventable deaths of newborns and under 5 years children with all countries aiming to reduce neonatal mortality to at least as low as 12 per 1,000 live births and under-5 mortality to at least to as low as 25 per 1,000 live births” has pushed the maternal and child health services in India for timely achieving this target [1, 2]. The concept of Universal Health Coverage (UHC) and equitable distribution of health services is expected to bridge the gap reflected by high variations in maternal and child health indices of our country [3]. Birthing is a natural phenomenon still fraught with danger for the mother and newborn, especially in cases of developing countries. Ensuring that labor and the first 24 hours postpartum are managed by a skilled health care provider is one of the keys to achieving the desired aim [4].

“The period from birth to 28 days of life in the neonatal period, and the infant in this period is a neonate or newborn baby” [5]. The days and weeks following childbirth, called the postnatal period, is a critical phase in the lives of mothers and newborn babies. This is the phase in life having the greatest risk of mortality and maximum potential for physical and neurocognitive developmental stress, which might be even long term. The State-Level Disease Burden Initiative Child Mortality Collaborators have revealed that the under-5-year mortality rate (U5MR) in India has decreased from 83.1 deaths per 1000 live births in 2000 to 42.4 deaths per 1000 live births in 2017 causing a 49% decrease, and the neonatal mortality rate (NMR) decreased from 38.0 deaths to 23.5 deaths per 1000 live births causing 38% decrease [6]. The variation of U5MR was 5.7 times between the different states and 10.5 times between 723 districts in 2017, whereas the neonatal mortality rate varied between 4.5 times (across the states) to 8.0 times (across the districts) [7].

The World Health Organization (WHO) stated that approximately 2.4 million neonates have died in 2019 and one-third of under 5 deaths also occur in neonatal time frame [8]. Neonatal hypothermia, defined by WHO as core body temperature less than 36.5ºc (97.7ºF). It is classified into three categories based on severity- mild (36.0 to 36.4 °C), moderate (32.0 to 35.9 °C), and severe (< 32 °C) [9–11]. Thermal control remains poor in newborns owing to immaturity of the thermoregulatory center and newborn become vulnerable to hypothermia especially premature babies, intrauterine growth retardation, and low birth weight (LBW) babies, and even normal-weight babies [12].

Neonatal hypothermia was associated with a five-times rise in mortality during the first 5 days of life [13]. Previous studies had also revealed that every one-degree Celsius decrease in a neonate’s body temperature increases the risk of mortality by 80% [12–14].

Deliveries conducted at home cause an unacceptably high proportion of maternal and perinatal mortality, especially in rural settings of India. The various clinical signs of neonatal hypothermia are “skin temperature less than 36.5ºc, hands, feet, abdomen is cold to touch, weak and lethargy, bluish extremities, slow heart rate and irregular respiration” [15, 16].

As for a newborn, the most frequent caretaker is their mothers, and it is the mother’s knowledge and practices that determine the future of the newborn. Traditional care practices at home, especially by caregivers and in the community affect maternal and newborn health inevitably [17].

Maintenance of the natural thermal environment is one of the most effective and cost-effective interventions to be practiced by everyone. Though the district Jyotiba Phule Nagar (Amroha) is a part of the developed region of Uttar Pradesh, yet, there may be certain gaps in the development of different sectors of the district including health. Even though few studies are assessing newborn care practices in Uttar-Pradesh, there was no study done in district Jyotiba Phule Nagar. The main aim was to assess the knowledge & practices regarding thermal protection & and find out the determinants responsible for that.

## Methods

A community-based cross-sectional study was conducted in Amroha District, previously known as Jyotiba Phule Nagar, which is one of the 75 districts of Uttar Pradesh state in northern India.

Study period: From 1st August 2018 to 31st July 2019 (12 months duration).

Study Population: The primary study population comprised women of reproductive age (15 to 49 years) who had delivered a live baby within the past 12 weeks before the conduct of this study (Recently Delivered Women). The 12-weeks limit was set with the purpose of resolving recall bias by the mother.

Sample Size Estimation: The required sample size for the survey among the mothers of children aged 0-12 weeks was calculated based on the standard formula for one point sample estimation:

$$\mathrm{Sample}\kern0.17em \mathrm{Size}=\frac{{\mathrm{Z}}_{1\hbox{-} \infty /2}^2\;\mathrm{P}\left(1\hbox{-} \mathrm{P}\right)}{{\mathrm{d}}^2}$$

To ensure coverage of the minimum required sample size for estimating various outcome indicators of the study, the value of ‘P’ (neonatal morbidity) is taken as 50.3% [10]. With the above assumption, the required sample size at 95% level of confidence with 5% of permissible error in the estimates, is worked out as n = 1.962 * 0.503 * 0.497 / 0.052 = 384. With consideration of a 10% non-response rate, the sample size was 427. The total sample of 427 households would be divided between the six blocks according to Probability Proportional to Size. Thus, in each block, several villages in each direction were selected for the survey with the assumption of 7 RDW per village to complete the required sample size.

### Study tool

A predesigned and pretested structured questionnaire in the form of multiple-choice questions was used as a study tool. It was developed to determine the knowledge and practices regarding thermal care of postnatal mothers/ caregivers on newborn care. That was divided into two 2 sections.

- Section A: Socio-Demographic information
- Section B: Knowledge and Practice questionnaire on newborn thermal care

The questionnaire had 12 items regarding Knowledge and Practices of mother/ caregiver about newborn care. Each correct response carried one mark; thus, the maximum possible score was 7 for knowledge and 5 for practices regarding thermal care. For each geographical area (blocks) knowledge and practices total score was calculated separately and categorized into three grades: Good, Fair, and Poor.

### Sampling Procedures

**Fig. 1:**
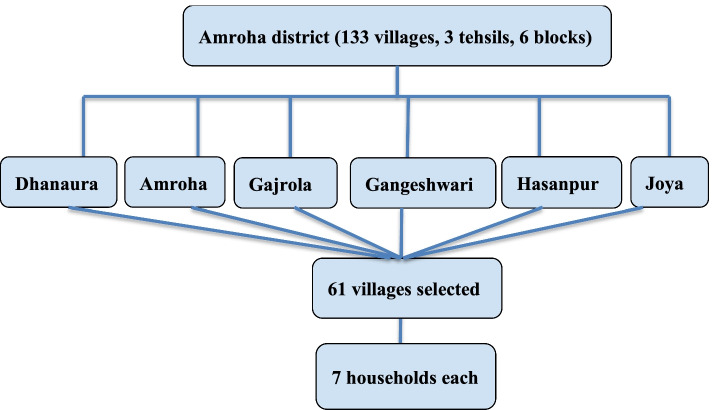
Sampling procedure of study participants

The district is divided into 1133 villages, 3 tehsils, and 6 blocks. Blocks are Dhanaura, Amroha, Gajrola, Gangeshwari, Hasanpur, and Joya. From each block most populous villages were selected, one near the CHC and 2nd farthest from the CHC and high priority was given to villages where ASHA and ANM were appointed. A total of 61 villages of 6 blocks were covered under the study (Fig. 1).

Procedure for Selection of Households- The number of households selected per village was fixed at 7. In each village, to select the required number of respondents, a systematic random sampling methodology was used. The field supervisor moved to the center of the village and selected the first household randomly. In the contacted households, it was verified whether the household had a child aged 0-12 weeks. If the child and the mother were present there, then the household was selected, and the structured questionnaire was canvassed. If not, the investigator moved to the immediate next household for a similar inquiry.

### Selection of Respondents

In each selected household, the child aged 0-12 weeks was identified, and the mother of the selected child was contacted for the interview. In a selected household, if more than one child was there, the mother of the youngest child would be contacted for the interview. The training was given for data collection to the Medical Social Workers for each block who were under a common field supervisor. Moreover, the overall data collection activities were supervised by the principal investigator.

Data analysis: The data were entered in Excel, critically analyzed, and tabulated using SPSS- 20 version software. Appropriate statistical tests of significance (Logistic regression) were applied to test and validate the findings of the study. Adjusted and unadjusted odds ratios (OR) and their 95% confidence intervals (CIs) were used as indicators of the strength of association. A p-value of 0.05 or less was used as a cut-off level for statistical significance.

## Result

Total 427 respondents were enrolled in the study from 6 blocks of the Amroha district. The maximum number of respondents were from Joya Block 98 (22.95%) followed by Amroha, Dhanaura, Gangeshwari, Hasanpur and the minimum number of respondents were from Gajrola Block 57 (13.3%). While assessing the socio-demographic variables of respondents more than half of the sample women 218 (51.1%) belonged to the 18 to 25 years age group, half 224 (52.5%) Hindu by religion, the majority of mothers, i.e., 251 (58.8%) in all the blocks never went to school & almost all, 421 (98.6%) were housewives by occupation. On the other hand, more than half of fathers 227 (53.2%) were laborers by occupation & illiterate 114 (26.7%) by education. Almost 258 (60.4%) respondents belonged to nuclear families & 264 (61.8%) respondents were from category IV socioeconomic class (Table 1). While assessing the respondent’s knowledge about thermal protection of baby, a wide variation was seen among the population of different blocks. Overall, 62.5% of the respondents were well aware of how to keep the baby warm after delivery. Maximum knowledge in these regards was found at Hasanpur followed by Gajrola block. 71.4% of respondents knew that baby should be dried soon after birth, 64.9% of the respondents had the idea of time to dry the baby, 69.6% of the respondents knew that baby should be wrapped soon after birth but only 27.4% of respondents mentioned the right time to wrap the baby. In all aforesaid parameters, maximum knowledge was found among mothers residing in the Joya block. Only 15.5% of the respondents knew what kind of cloth should be used to wrap the baby. Among all Knowledge components, Maximum knowledge was observed about the right time when to give the first bath to the baby. 90.6% of mothers had correct knowledge in this concern (Table 2). Promoted thermal protection to improve the “warm chain” for newborns, is an essential component of essential newborn care (ENC). ENC to prevent neonatal hypothermia (temp< 36.5), a leading cause of neonatal mortality. Out of the total, 53.6% of the respondents reported that they had placed the baby in skin contact on belly/chest after birth and this practice was found to be most prevalent in Gajrola followed by Hasanpur block. 44.5% of respondents had placed the baby at the right time in skin contact on the belly/chest, 60.2% of respondents wiped the baby within the stipulated time after delivery but only 20.0% of caregivers wrapped the baby with a cloth after delivery within the expected time. 82.4% of caregivers claimed that they had given the first baby bath at the recommended time. Block-wise variation was observed in different practices. Thermal care was best practiced at the Gajrola block except for one domain, i.e., wrapping of the baby in cloth after delivery. Worst practice performance was found at Joya block for most of the thermal care practices (Table 3). While assessing the determinants responsible for the knowledge regarding Thermal Protection, age of mother, child’s mother religion, education, occupation was found significantly associated with the knowledge of the respondents. Good knowledge found in Mothers of age more than 35 years, belongs to non-Hindu religion, nonworking, having above 10th education level, and belongs to either socioeconomic status I or II and from nuclear family. The effect of a mother’s age, religion, education, and occupation on knowledge regarding thermal protection is found statistically significant (Table 4).

**Table 1:** Respondents/Mothers population distribution (n 427)

Sociodemographic Variables	Response n (%)
**Community Blocks**	
Dhanaura	64 (14.99)
Amroha	83 (19.44)
Gajrola	57 (13.35)
Gangeshwari	63 (14.75)
Hasanpur	62 (14.52)
Joya	98 (22.95)
**Age groups**	
18-25 years	218 (51.1)
26-30 years	176 (41.2)
31-35 years	25 (5.9)
>35 years	8 (1.9)
**Child’s Mother religion**	
Hindu	224 (52.5)
Muslim/Sikh/Other	203 (47.5)
**Child’s Mother education**	
≤10^th^	294 (68.9)
>10^th^	33 (31.1)
**Child’s Mother occupation**	
Housewife	421 (98.6)
Working	06 (1.4)
Socio-economic status*	
I	5 (1.2)
II	19 (4.4)
III	67 (15.7)
IV	264 (61.8)
V	72 (16.9)
**Type of family**	
Nuclear	258 (60.4)
Joint	169 (39.6)

**Table 2:** Percentage of respondents having correct knowledge about thermal protection

Thermal protection-Variable	Respondents having correct knowledge						Total^**a**^
	Dhanaura	Amroha	Gajrola	Gangeshwari	Hasanpur	Joya	
Methods to keep baby warm after delivery	69.8%	63.95%	73.7%	60.3%	74.2%	43.9%	62.5%
Should baby be dried after birth	73.4%	73.5%	77.2%	55.6%	51.6%	87.8%	71.4%
Time to dry the baby	62.5%	67.5%	77.2%	47.6%	48.4%	78.6%	64.9%
Should baby be wrapped after birth	75.0%	75.9%	77.2%	41.3%	48.4%	87.8%	69.6%
Time to wrap the baby	28.1%	34.9%	10.5%	13.2	5.4%	54.1%	27.4%
Kind of cloth should be used to wrap the baby	42.2%	16.9%	8.7%	19%	9.7%	3.1%	15.5%
Time to give first bath to baby	98.4%	96.4%	94.7%	77.8%	83.9%	90.8%	90.6%

^a^Percentage of total study population

**Table 3:** Percentage of respondents/mothers/caregivers with correct Thermal Protection related Newborn Care Practices (immediately after recent delivery)

Variable	Respondents having correct practice						Total^**a**^
	Dhanaura	Amroha	Gajrola	Gangeshwari	Hasanpur	Joya	
After birth baby was placed in skin contact on belly/chest	51.9%	58.7%	68.4%	49.2%	66.1%	37.1%	53.6%
When was the baby placed in skin contact on belly/chest	51.6%	41.0%	56.1%	40.8%	57.7%	32.9%	44.5%
When was the baby wiped (dried) after delivery	61.9%	58.7%	75.4%	42.9%	40.0%	76.5%	60.2%
When was the baby wrapped with cloth after delivery	25.0%	31.3%	8.8%	9.5%	4.8%	38.8%	22.0%
How long after birth was baby bathed for first time	89.1%	91.6%	87.7%	68.3%	72.6%	82.7%	82.4%

^a^Percentage of total study population

**Table 4:** Determinants of Good Knowledge about thermal protection among respondents

Variable	Thermal Protection (Have Good knowledge n=237)	
	Unadjusted OR (95%CI)	Adjusted OR (95% CI)
**Age of mother**		
18-25 years^Ref^	1	1
26-30 years	1.0 (0.7-1.5)	0.9 (0.7-1.8)
31-35 years	0.9 (0.7-1.6)	0.8 (0.6-1.8)
>35 years	1.4 (1.1-2.2)	1.3 (1.1-2.1)
*p* value*	**0.037**	
**Child’s Mother religion**		
Hindu^Ref^	1	1
Muslim/Sikh/Other	1.3 (1.2-2.1)	1.3 (1.1-2.2)
*p* value	**0.032**	
**Child’s Mother education**		
≤10^thRef^	1	1
>10^th^	1.8 (1.6-2.7)	2.1 (1.8-2.8)
*p* value	**0.028**	
**Child’s Mother occupation**		
Housewife ^Ref^	1	1
Working	0.7 (0.4-1.3)	0.7 (0.5-1.2)
*p* value	**0.031**	
**Socio-economic status**		
I^Ref^	1	1
II	1.0 (0.8-1.2)	1.0 (0.9-1.1)
III	0.8 (0.5-1.2)	0.6 (0.4-1.2)
IV	0.8 (0.7-1.3)	0.7 (0.5-1.1)
V	0.7 (0.5-1.1)	0.5 (0.4-1.2)
*p* value	**0.059**	
**Type of family**		
Nuclear^Ref^	1	1
Joint	0.9 (0.7-1.4)	0.8 (0.5-1.3)
*p* value	**0.052**	

*P-value is of unadjusted odds ratio

Mothers of age more than 35 years, non-working, having above 10th education level, belong to socioeconomic status I, from a joint family, and preferred institutional delivery are found to be effective determinants for newborn thermal care practices. Among these determinants, good thermal practices were significantly associated with the age of the mother, mother’s education, socioeconomic status, and type of family only (Table 5) Table 6.

**Table 5:** Determinants of Good Newborn Care Practices among respondents

Variable	Good Thermal Protection Practices(N=179)	
	Unadjusted OR (95%CI)	Adjusted OR (95% CI)
**Age of mother**		
18-25 years^Ref^	1	1
26-30 years	0.7 (0.5-1.1)	0.8 (0.6-1.1)
31-35 years	1.0 (0.8-1.2)	1.2 (1.1-1.7)
>35 years	1.3 (1.1-2.2)	1.3 (1.2-1.9)
*p* value*	**.028**	
**Child’s Mother religion**		
Hindu^Ref^	1	1
Muslim/Sikh/Others	1.0 (0.8-1.4)	0.9 (0.7-1.3)
*p* value	**0.059**	
**Child’s Mother education**		
≤10^thRef^	1	1
>10^th^	2.0 (1.7-2.5)	1.9 (2.1-2.2)
*p* value	**0.038**	
**Child’s Mother occupation**		
Housewife ^Ref^	1	1
Working	0.7 (0.5-1.6)	0.7 (0.6-1.2)
*p* value	**0.098**	
**Socio-economic status**		
I^Ref^	1	1
II	0.6 (0.4-1.2)	0.6 (0.5-1.3)
III	0.5 (0.4-1.1)	0.5 (0.4-1.1)
IV	0.5 (0.3-1.2)	0.5 (0.4-1.2)
V	0.4 (0.3-1.3)	0.4 (0.3-1.4)
*p* value	**0.023**	
**Type of family**		
Nuclear^Ref^	1	1
Joint	1.4 (1.2-1.7)	1.4 (1.3-1.8)
*p* value	**0.041**	
**Type of delivery**		
Home Ref	1	1
Institutional	1.2 (1.1-1.7)	1.2 (1.1-1.6)
*p* value	**0.028**	

*P-value is of unadjusted odds ratio

**Table 6:** Gap analysis in between Knowledge & Practices among respondents

Variable	Thermal Protection Mean Score (SD)	
	Knowledge	Practices
Total	5.18 (3.24)	3.28 (2.21)
**Block**		
Amroha	5.86 (3.86)	3.12 (1.24)
Dhanaura	5.48 (3.36)	4.04 (1.94)
Gajrola	6.08 (4.98)	3.92 (1.74)
Gangeshwari	3.66 (1.24)	2.81 (1.14)
Hasanpur	3.96 (1.58)	3.86 (1.28)
Joya	5.98 (3.33)	3.96 (1.62)
**Age of mother**		
18-25 years	5.36 (3.72)	3.98 (1.84)
26-30 years	5.88 (3.16)	2.96 (1.36)
31-35 years	4.48 (2.68)	4.02 (2.44)
>35 years	6.16 (4.84)	4.42 (2.24)
**Child’s Mother religion**		
Hindu	5.08 (3.64)	3.86 (1.64)
Muslim	6.14 (4.56)	3.02 (1.92)
Sikh	6.26 (4.84)	4.12 (2.84)
**Child’s Mother occupation**		
Housewife	5.88 (3.54)	3.98 (1.98)
Working	3.16 (1.86)	3.72 (1.62)
**Child’s Mother education**		
<10^th^	3.36 (1.78)	3.78 (2.24)
>10^th^	5.12 (3.24)	4.02 (1.74)
**Type of family**		
Nuclear	5.28 (3.92)	3.64 (1.54)
Joint	4.96 (2.88)	4.08 (2.24)
**Socio-economic status**		
I	6.48 (4.14)	4.40 (2.06)
II	6.42 (4.36)	4.18 (2.18)
III	5.86 (3.98)	3.78 (1.44)
IV	5.32 (3.44)	3.22 (1.86)
V	4.12 (2.58)	3.02 (1.32)

**Fig. 2:**
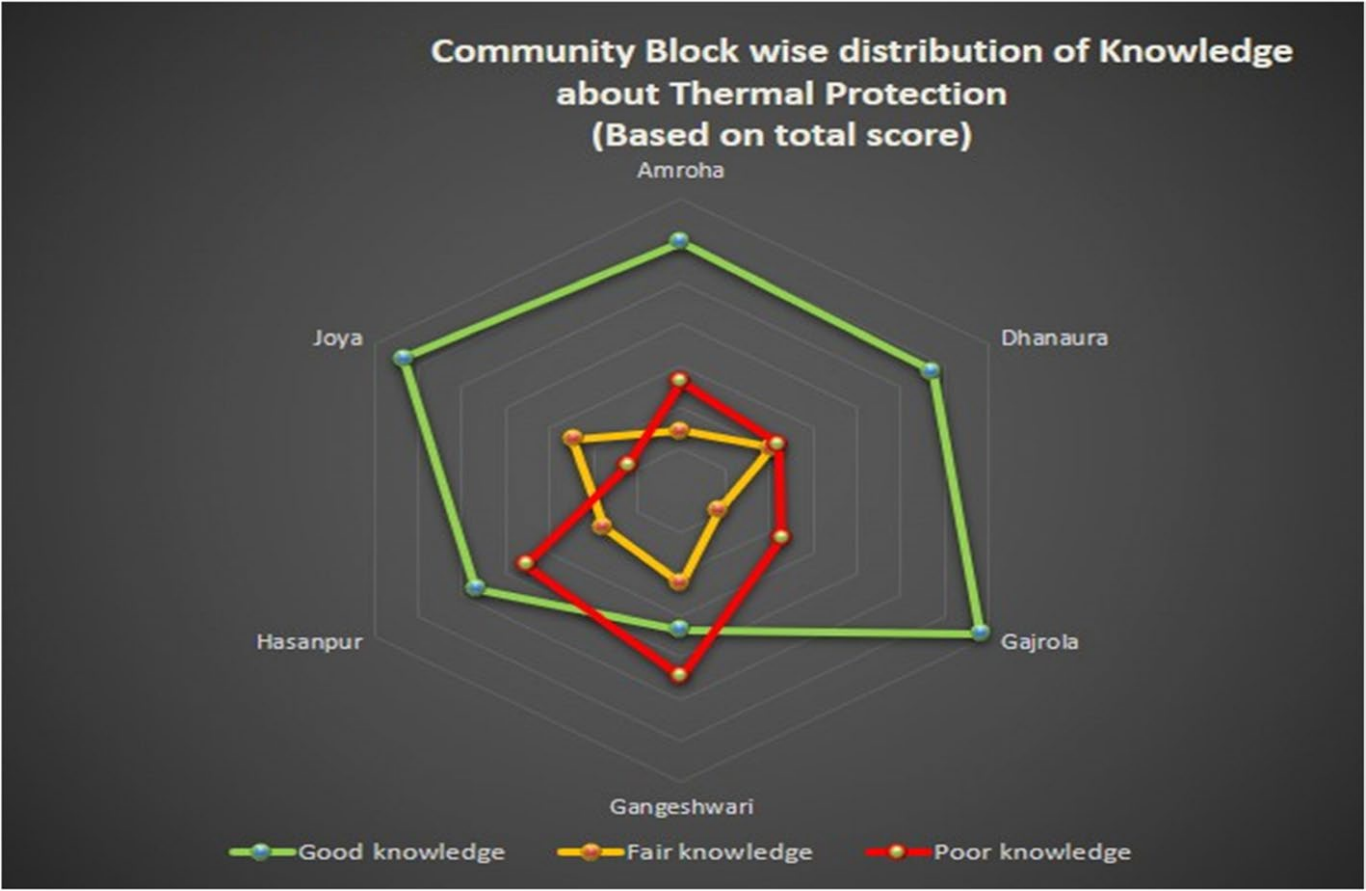
Community block wise distribution of knowledge about thermal protection

**Fig. 3:**
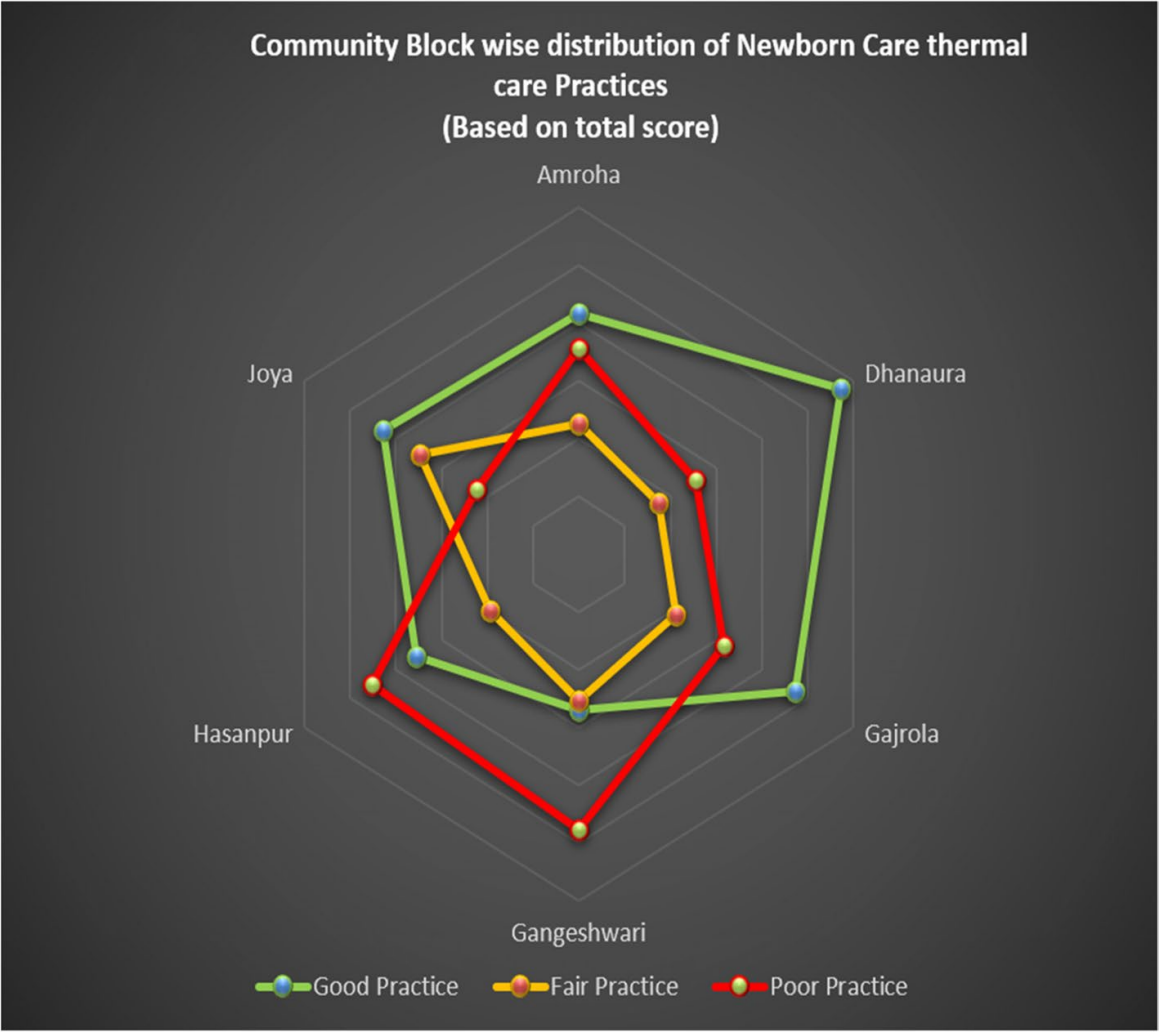
Community wise distribution of newborn care thermal practices

Overall knowledge about thermal protection is good in the majority of 237 (55.5%) of the respondents. In the population pool having good knowledge, the maximum contribution was from Joya and the minimum from the Gangeshwari block. Overall, 25.9% of respondents had a poor level of knowledge and the worst condition in this pool was found in the Gangeshwari block (Fig. 2). The overall majority (41.9%) of respondents had good newborn practices score while 24.1% and 34.0% of respondents had fair and poor newborn practices score respectively. More than half of the population scored a good level of practice at Dhanaura block, whereas almost half of the population was found under a poor score of practices at Gangeshwari block (Fig. 3).

## Discussion

Incidence of hypothermia in newborns in India varies from about 11 percent of all newborns to as many as 45% of newborns in home settings. Behavioral risk factors like not drying or wiping the newborn immediately after birth, bathing the newborn soon after birth are among the major risk factors for neonatal hypothermia, while skin-to-skin contact can be an important measure to prevent hypothermia [18, 19]. We have studied maternal behavioural risk factors associated with neonatal hypothermia in this study. While assessing the knowledge on thermal protection of babies, it was found that overall knowledge about thermal protection is good in the majority 237 (55.5%) of the respondents & fair in 79 (18.5%) of respondents. In a study done in Northern India about knowledge on prevention of heat loss, 45% of mothers had adequate knowledge of drying the baby immediately. Association of the concept of bathing the newborn as a cause of hypothermia was observed in 15% of mothers. 45% of mothers knew that not wiping the baby or not drying after birth may cause hypothermia. Only 54% of mothers knew covering the baby with a warm blanket or clothes in multiple layers to prevent heat loss [20]. Similar findings were reported in a study from Dhaka, Bangladesh, explained that babies were typically bathed soon after birth to purify them from the birthing process [21]. In Nepal, less than half of newborns were wrapped within the first 10 minutes after birth and almost all of them were bathed within minutes or hours after birth [22, 23]. A study was done by Tumla Shrestha, Saraswoti Gautam Bhattarai, Kalpana Silwal in Nepal 2013 [24] about the knowledge of a postnatal mother on the care of a newborn, majority 76 (76%) answered as keeping the newborn warm, 50 (50%) said bathing and cleaning and 82 (82%) of respondents said wrapping with warm clothes & nine (9%) have knowledge in delay bathing to keep newborn warm. Studies mostly from South Asia include insufficient heating of the birthplace, placing the uncovered newborn on the ground or other cold surfaces, delayed wrapping, and early bathing all contributing to hypothermia [24–26]. The findings of this study except the timing of bathing after birth are similar to the studies from other parts of India, Nepal, and Bangladesh which reflects on the similar socio-cultural milieu in the Indian sub-continent. Wiping was correctly practiced by 60.2% of mothers, and 82.4% were delayed the first baby bath. Wiping the babies is practiced by 31% of mothers to protect from hypothermia, and many thought baby bath should not be done or must be postponed [27]. Hypothermia during transfer is also an aggravating factor that should be avoided. 54% of the mother knew covering the baby in layers of cloth and blankets and only 3% of mothers were aware of KMC to protect the baby from hypothermia during transport [28–30]. Our study shows that an increasing level of maternal education and the specific religious group were associated with better thermal protection knowledge and practice of the newborn. Similar findings have been reported in the study from Nepal. Higher educational status may be associated with an increased level of awareness regarding good practices of thermal protection. Also, mothers with higher education and may be able to resist harmful cultural practices regarding thermal protection prevalent in the community [31]. Increasing age is likely to be associated with higher parity, which may be associated with more exposure to thermal protection knowledge and make the practices better during previous pregnancies and childbirth episodes. This finding is corroborated by a study done in Uganda [32].

Working women though had poor knowledge of measures of prevention of hypothermia in the newborn in our study. It may be explained by the fact that the district in which this study was done is one of the less developed districts in India. Thus, it may be possible that only women with very poor socioeconomic status and limited, or no education are working to feed their families- likely in the informal sector among the study participants.

Higher socioeconomic status, higher levels of education and delivery in an institution are inter-related and well documented [33]. All these factors were significantly associated with better practices regarding the prevention of hypothermia among the newborns in the mothers in our study. Mothers living in joint families had significantly more correct practices concerning hypothermia prevention in our study. This may be due to the transmission of information regarding newborn rearing practices among women living in joint families — some of whom might have had previous childbirth in institutional settings or have acquired correct information during previous pregnancies or childbirth. It should also be noted that even within the same district, the knowledge, and practice regarding good newborn care practices preventing hypothermia are not uniform across the sub-divisions within the districts (Block/ tehsils). While Gangeshwari and Hasanpur blocks had the lowest score both in the knowledge component; Gangeshwari and Amroha had lower scores in the practice component. The differences may be due to different levels of industrialization and urbanization in these blocks and also because of diversity in religious and social composition across these blocks. The knowledge-practice gap analysis in our study showed that residents of Amroha block had better knowledge but lack practice, on the contrary Hasanpur residents had better newborn thermal practice without good knowledge. Similarly, the practice component was low among Muslim mothers despite having good knowledge. On the contrary, working mothers managed to provide better thermal care practices to newborns despite low knowledge. However, our study had few limitations as well. Firstly, the study was conducted in a district of North India which is relatively underdeveloped. Thus, the findings cannot be generalised to other districts of North India and South India. The mothers who refused to participate in our study were not included in the analysis part, so non -responses or drops were unaccounted for. The interview was conducted in the local language and the responses were marked by the Medical Social workers which may lead to bias due in the interpretation of responses despite prior training.

Few strengths of our study were, the inclusion of diverse group of mothers belonging to different religions, castes, and socioeconomic statuses. This study also surfaced the disparity in knowledge and practice domain within close geographical locations as in the district itself. Few lessons learned from our study were, just the knowledge does not suffice when it comes to newborn care and practicing newborn care practices without proper knowledge may not prove to be effective enough for the newborn.

## Conclusion

The findings of the study provide an insight into the existing knowledge and necessitate a need for quantitative studies in the study area to access knowledge & practices related to the thermal protection of newborns. There is a need for improving community awareness to promote institutional deliveries and improve the health system to satisfy the demands of birthing women. When home birth is inevitable, families should be encouraged to engage skilled birth attendants to provide better newborn care. Understanding and addressing community-based practices on hypothermia, prevention, and management might help to improve newborn survival in resource-limited settings. Infants born small or prematurely are recognized as needing more intense thermal protection. Health education on essential newborn care practices should be integrated into routine antenatal services and re-emphasized in the postnatal period to help improve maternal knowledge towards thermal care practices.

## Supplementary Information

**Additional file 1.**

## Data Availability

Study questionnaire provided as supplementary file. Study findings can be shared on reasonable request.

## Abbreviations

ANM: Auxiliary Nurse Midwife
ASHA: Accredited Social Health Activist
CHC: Community Health Centre
CI: Confidence Interval
ENC: Essential Neonatal Care
LBW: Low Birth Weight
MS: Microsoft
OR: Odds Ratio
RDW: Recently Delivered Women
SDGs: Sustainable Development Goals
WHO: World Health Organization
