# Supplementary material for: Newborn thermal care in western Uttar Pradesh — gap analysis between knowledge and practices
Source: Arch Public Health. 2022 Feb 16;80:55. doi: 10.1186/s13690-022-00809-2 (PMC8848645; doi:10.1186/s13690-022-00809-2)
Supplement: Supplementary file 1 — Additional file 1. [file 13690_2022_809_MOESM1_ESM.doc]

***NEONATAL CARE PATTERN IN RURAL AMROHA***

***EXPLORE THE GAPS & POTENTIAL IMPROVEMENT OPTIONS***

**SECTION A**

Knowledge and Pattern of Essential Newborn Care (Mothers/Caregivers)

Date: / / Block Name:

Village Name: Village Code:

Respondent Name: Relation with child:

Head of family: Household No:

**General Information**

1. Respondents age years

2. Year of married life:

3. Child’s mother occupation

a)Farmer b)Daily laborer c) Merchant d)Housewife e)Govt.employer f)Other (specify) ___________

4. Child’s mother education

a)Illiterate b)Just literate c)Primary d)Secondary e)High school f)Intermediate g)Graduate h)Post Graduate

5. Child’s mother religion

a) Hindu b) Muslim c) Sikh d) other__________

5.1 If Hindu her caste: OBC/SC/ST/OTHERS

6. What is the main occupation of Child’s father?

a)Farmer b)Daily laborer c)Merchant

d)Govt.employer e)Other (specify) ___________

7. What is Child’s father education?

a)Illiterate b)Just literate c)Primary d)Secondary e)High school f)Intermediate g)Graduate h)Post Graduate

8. Family income per capita: _________________________

8.1 Socio-economic status of family: I / II / III / IV / V

9. Type of family: Nuclear / Joint / Three generation

9.1 Family size:

10.Child’s Birth Date

11. Type of delivery Home/ Institutional

If home

Delivery conducted by whom ASHA/ANM/TBA/Doctors/Family member/Others (specify)

Reason of home delivery Preferred to deliver at home

Cost too much

Too far/ no transportation

Not necessary

Not customary

Delivered on the way to health facility

Other

**Knowledge of Mother/ Caregiver About Newborn Care**

| **Thermal Protection** | | | |
| --- | --- | --- | --- |
| 101 | | How should you keep your baby warm after delivery? | Skin to skin contact  Wrapped the baby in a cloth  Other(specify) |
| 102 | | Should we dry the baby soon after birth | Yes/No/Don’t Know |
|  |  | Time to dry the baby | within 10 minutes after birth  within 10-30 minutes after birth  after 30 – 60 minutes  only after the bath |
| 103 | | Should we wrap the baby soon after birth  If yes | Yes/No/Don’t Know |
|  |  | Time to wrap the baby | within 10 minutes after birth  within 10-30 minutes after birth  after 30 – 60 minutes  only after the bath |
|  |  | What kind of cloth should be used to wrap the baby? | old unwashed cloth  old washed cloth  others  new unwashed cloth  new washed cloth  unknown |
| 104 | | How long should you take before you give your baby the first bath after delivery? | Soon after delivery  1 to 6 hours  between 6 -24 hours  More than 24 hours  Don’t know |
| **Cleanliness and Cord Care** | | | |
| 105 | What type of instrument should be used for umbilical cord cut? | | Scissors  Sickle  any household sharp  Used shaving blade washed with water  sterilized new blade  Any other (specify) |
| 106 | After cord cut the stump should be tied | | Yes/No/ Don’t know |
|  | If yes with what | | Sewing thread  nylon thread  sterilized thread  string  cord clamp  any other (specify) |
| 107 | What should we do after cord cut for cord care | | Left open uncovered  covered with turmeric  covered with cloth  covered with cloth and then applied powder  application of cow dung  tip burnt with castor lamp  tip burnt and powder applied application of antiseptic cream/lotion  application of mustard oil  other (specify) |
| 108 | If the umbilical stump is soiled with baby’s urine or faeces how would you clean it? | | Clean with water  clean with saliva  Apply alcohol or spirit  other (specify) |
| 109 | After cleaning your baby’s soiled umbilical stump, should any substances be applied to it? | | Yes/No/ Don’t Know |
|  | If yes, what material should be applied on your baby’s umbilical stump? | | Left open uncovered  covered with turmeric  covered with cloth  covered with cloth and then applied powder  application of cow dung  tip burnt with castor lamp  tip burnt and powder applied  application of antiseptic cream/lotion  application of mustard oil  other (specify) |
| **Breastfeeding** | | | |
| 110 | How soon after delivery you should initiate breastfeed your baby? | | Within 1 hr  Between 1–4 hours  Between 4–24 hours  Between 1–3 days  Between 3–7 days  After 7 days  Don’t Know |
| 111 | Should any fluid/feeds give to baby before breastfeeding for the first time? | | Yes/No/Don’t Know  If yes what (spacify)……………… |
| 112 | Should colostrums feed to baby? | | Yes/no/don’t know |
| 113 | How often should you breastfeed your baby? | | On demand  According to timetable (frequency)  Other (specify) |
| 114 | How long should you exclusively breastfeed your baby (in months)? | | ...................... |
| **Immunization** | | | |
| 115 | | Does your baby require any vaccination at birth? | Yes/No/Don’t Know |
| 116 | | Why do we give vaccines to the baby after birth? | To prevent diseases  Don’t know  Other (specify) |
| 117 | | What vaccines should your baby received at birth? | BCG  OPV  Don’t know  Other (specify) |
| **Eye Care** | | | |
| 118 | | Are you aware of any signs that would make you know your baby has an eye infection? | Eye discharge  Reddening of eye  Swollen eye  Other (specify) |
| 119 | | What should be you do if any of above symptom appear | Apply Kajal/Surma  Any other home remedy (specify) ………………  Contact doctor/HW  Do Nothing |

**Newborn Care Practices**

*Now I would like to ask you about newborn care practices immediately after recent delivery*

| 120 | After birth, was the baby placed in skin contact on your belly/chest?  If Yes  When was the baby placed in skin contact on your belly/chest? | Yes/No/Don’t Know |
| --- | --- | --- |
|  |  | Immediately after birth  After cord cut  After baby dried  only after the bath  Other (Specify) ____________________ |
| 121 | When was the baby wiped (dried) after delivery? | Within 10 minutes after birth  Between 10-30 minutes after birth  Between 30 – 60 minutes  Not wiped  Don’t know |
| 122 | When was the baby wrapped with cloth after delivery? | Within 10 minutes after birth  Between 10-30 minutes  Between 30 – 60 minutes  Not wrapped  Don’t know |
| 123 | Did your baby cry or breathe easily immediately after birth?  If no  What was done to help the baby cry or breathe at the time of birth, if anything? | Yes/No/Don’t Know |
|  |  | Rubbed/massaged  Dried  Mouth cleared  Nothing  Other (Specify) __________  Don’t know |
| 124 | Who took these measures to help the baby cry or breathe? | Doctor  ANM  ASHA  Traditional birth attendant  Relative/Family member  Other (specify)__________ |
| 125 | Where was the baby placed immediately after delivery? | On the floor  On the mother’s belly/chest  Beside the mother  With someone else  On newborn bed/table  Other (specify)________________  Don’t know |
| 126 | Was there a person who took care of the newborn while you were delivering the placenta? | Yes/No/Don’t Know/Can’t Remember |
| 127 | Who took care of the newborn? | Doctor  ANM  ASHA  Traditional birth attendant  Relative/Family member  Other (specify)________________ |
| 128 | What was used to cut the cord? | Scissors  Sickle  any household sharp  Used shaving blade washed with water  sterilized new blade  Other (specify) ___________  Don’t Know/Can’t Remember |
|  | If answer is other than sterilized new blade then  Was the instrument used to cut cord boiled prior to use? | Yes/No/Don’t Know/Can’t Remember |
| 129 | What was used to tie the cord? | Sewing thread  nylon thread  sterilized thread  string  cord clamp  Cord was not tied  Other (specify) ___________  Don’t know |
| 130 | If answer is other than sterilized thread then  Was the tie/string used to tie the cord boiled prior to use? | Yes/No/Don’t Know/Can’t Remember |
| 131 | Was anything applied to the cord immediately after cutting (and tying)? | Yes/No/Don’t Know/Can’t Remember |
|  | If yes then  What was applied to the cord just after cutting the cord? | Left open uncovered  turmeric  covered with cloth  covered with cloth and then applied powder  Ash/soil  cow dung  tip burnt with castor lamp  tip burnt and powder applied  antiseptic cream/lotion  oil  Butter  Other (specify) _______________________  Don’t know |
| 132 | How much did (NAME) weigh at birth?  ***.*** | Weight from card in kilograms _____.______  Weight from recall______.______  Baby not weighed  Don’t know |
| **For mothers who reported perceiving their babies to be small or reported a birth weight of less than 2.5K g (Q 133- 139)** | | |
| 133 | Since your baby was small, did you receive extra visits or care for your baby? (Eg. more visits than normal, referral to hospital) | Yes/No/Don’t know |
| 134 | Since your baby was small, what extra care did you give to your baby? | More frequent breastfeeding  Skin-to-skin contact  Nothing  Other (Specify) _______________________  Don’t know |
| 135 | Did you put your baby in KMC position? | Yes  No  If no then skip to Q …. |
| 136 | When did you first put your baby in KMC position? | Immediately after birth  In the first 24 hours after delivery  In the first three days after delivery  In the first week after delivery  After the first week of delivery |
| 137 | How many days was (name) in KMC position, at least part of the day, until you no longer put (name) in KMC position? | ____________________days  Don’t remember |
| 138 | During the days that (name) was in KMC position, how long did you keep him/her in KMC position? | The whole day (daytime and night time)  About half a day (only daytime or only nighttime)  About quarter of a day (half daytime or half nighttime)  Less than a quarter of a day  Don’t remember |
| 139 | Any other home level indigenous practice you did for survival and rehabilitation of child. | Specify …………………………………………………………………………………………………………………………………………………………………………………………………………… |
| 140 | In the first two days of life was (name) breastfed or cup-fed? | Breastfed only  Cup fed with breast milk only  Cup fed with non-breast milk products only  Both breastfed and cup fed with breastmilk  Both breastfed and cup fed with non-breastmilk  Bottle fed with breast milk only  Bottle fed with non-breast milk products only  Other  Don’t know |
| 141 | How often was (name) breastfed or cup fed?  Probe for number of times fed during the day and number of times fed during the night | number of times  ………….(During Daytime)  ………….(During Night) |
| 142 | How long after birth was (NAME) bathed for the first time? | Soon after delivery  1 to 6 hours  More than 6 hours but less than 24 hours  More than 24 hours (specify days) ________  don’t know |
| 143 | Was the baby put to the breast before the placenta was delivered? | Yes  No  Don’t know |
| 144 | How long after birth did you first put (NAME) to the breast? | Within 1 hr  Between 1–4 hours  Between 4–24 hours  Between 1–3 days  Between 3–7 days  After 7 days  Don’t know  Never breastfed |
| 145 | Did you squeeze out and throw away the first milk (colostrums)? | Yes/ No |
| 146 | After delivery any fluid/feeds was given to drink before breastfeeding for the first time | Yes/ No/Don’t know |
|  | If yes  What was (NAME) given to drink? | \| Milk (other than breast milk \| \| --- \| \| Plain water \| \| Sugar or glucose water  Honey \| \| Fruit juice \| \| Infant formula \| \| Tea/infusions \| \| Fresh Butter \| \| Ghutti \| \| Other (Specify) ____________ \| \|  \| |
| 147 | What did you (or the birth attendant) do to keep (NAME) warm following delivery?  Multiple responses possible | Dried the baby  Wrapped the baby with clean cloths  Put baby beside the mother  Kept the baby on bare skin to skin contact  Bathed in warm water  warmed delivery room  Other (specify) ___________________  Nothing done  Don’t know |
